# Technology Use Among Older Adults to Manage their Health During a Global Pandemic

**DOI:** 10.1093/geroni/igab046.2741

**Published:** 2021-12-17

**Authors:** Gashaye M Tefera, Erin Robinson, Geunhye Park

**Affiliations:** University of Missouri, Columbia, Missouri, United States

## Abstract

Risk of severe COVID-19 illness increases with age, and older adults are more likely to be hospitalized and die from COVID-19 and related complications as compared to their younger counterparts. This reality, combined with pandemic-related lockdown and social distancing policies, has increased in-home isolation for older adults. This includes cancelling in-person healthcare appointments and conducting many appointments via tele-health. As older adults have had to quickly pivot to learning new technologies, little is known about their experiences with navigating virtual healthcare during the pandemic. Therefore, this qualitative study aims to address that gap. One-on-one interviews (N=29) were conducted with older adults (Mean age=71.5; 86% female) via phone/Zoom. Participants were asked about their healthcare experiences during the pandemic and the role technology played. Interviews were transcribed and thematically analyzed using Nvivo12 software. Findings demonstrate that participants used technology to schedule medical appointments, engage in virtual visits with their providers, set reminders to take medications, and undertake their daily exercise routine. Post-lockdown, some participants preferred in-person visits due to the nature of their diagnosis, personal preference, or unfamiliarity with the needed technology. Older adults encountered challenges including cancelled appointments, miscommunication with providers, and lack of skill to use technologies. Cancellation of appointments and postponement of treatments affected the health of some of the participants. Implications of this research can inform tele-health approaches with older patients, as well as provider communication and coordination of care. Leveraging technology for preventative health approaches can also assist older adults in ongoing health maintenance and promote well-being.

